# A human *ex vivo* coculture model to investigate peritoneal metastasis and innovative treatment options

**DOI:** 10.1515/pp-2021-0128

**Published:** 2021-07-27

**Authors:** Dina Mönch, Jana Koch, Annika Maaß, Nicole Janssen, Thomas Mürdter, Philipp Renner, Petra Fallier-Becker, Wiebke Solaß, Matthias Schwab, Marc-H. Dahlke, Hans J. Schlitt, Tobias Leibold

**Affiliations:** Dr. Margarete Fischer-Bosch Institute for Clinical Pharmacology, Stuttgart, Germany; University of Tübingen, Tübingen, Germany; Department of General and Visceral Surgery, Robert-Bosch-Hospital, Stuttgart, Germany; University Medical Center Regensburg, Regensburg, Germany; Institute of Pathology, University of Tübingen, Tübingen, Germany; Departments of Clinical Pharmacology, Pharmacy, and Biochemistry, University of Tübingen, Tübingen, Germany

**Keywords:** colorectal cancer, *ex vivo* model, HIPEC, peritoneal metastasis, peritoneum

## Abstract

**Objectives:**

Peritoneal metastasis (PM) is commonly observed in patients with colorectal cancer (CRC). The outcome of these patients is poor, with an average survival of only six months without therapy, which requires a better understanding of PM biology and new treatment strategies.

**Methods:**

We established and characterized a human *ex vivo* peritoneal model to investigate the mechanisms of peritoneal seeding and possible treatment options. For this, CRC cell lines and patient-derived tumor organoids were cultured together with human peritoneum to investigate the invasion of malignant cells and the effects of local chemotherapy.

**Results:**

Fresh human peritoneum was cultured for up to three weeks in a stainless steel ring system, allowing for survival of all peritoneal structures. Peritoneal cell survival was documented by light microscopy and immunohistochemical staining. Further, immunohistological characterization of the tissue revealed CD3-positive T-lymphocytes and vimentin-positive fibroblasts within the peritoneum. In addition, extracellular matrix components (collagens, matrix metalloproteinases) were localized within the tissue. Coculture with CRC cell lines and patient-derived CRC organoids revealed that cancer cells grew on the peritoneum and migrated into the tissue. Coculture with CRC cells confirmed that hyperthermal treatment at 41 °C for 90 min significantly enhanced the intracellular entry of doxorubicin. Moreover, treatment with mitomycin C under hyperthermic conditions significantly reduced the amount of cancer cells within the peritoneum.

**Conclusions:**

This human *ex vivo* peritoneal model provides a stringent and clinically relevant platform for the investigation of PM and for further elucidation of possible treatment options.

## Introduction

In 2020, colorectal cancer (CRC) ranked among the top five leading cancer types, both in estimated new cases and cancer-related deaths [1, 2]. Depending on the (histological) subtype, up to half of all patients develop metastasis via two major routes of spreading: (i) Delivery of single tumor cells via the bloodstream and/or lymphatic system to the liver or lung; and (ii) locally and directly disseminated single tumor cells, small cell clumps or complete glandulae, that get shed off e.g. during surgery, throughout the peritoneal cavity that induce peritoneal metastasis (PM) [3, 4]. Patients with PM alone or in combination with other metastatic sites, such as the liver and lung, have a dismal prognosis compared to that of patients without PM [5]. Thus, for a long time, PM was regarded as a terminal, incurable disease and was mainly treated with palliative chemotherapy and management of complications, such as obstruction, bleeding, or perforation [6].

Two decades ago, peritoneal cytoreductive surgery (CRS) and hyperthermic intraperitoneal chemotherapy (HIPEC) started to improve the survival of patients with PM mostly using mitomycin C (MMC), oxaliplatin, or doxorubicin. However, these therapies are limited to a stringently selected patient cohort and are associated with high morbidity, especially when performed outside of specialized centers [7]. However, data on clinical outcome for all cytoreductive treatments must still be considered controversial, particularly when real-life endpoints are assessed vs. best systemic chemotherapy. Recent studies even propose that CRS alone should be the therapeutic strategy with curative intent for colorectal PM [8]. However, one of the few randomized, prospective trials by Verwaal and others showed an increased median survival from 12.6 to 22.3 months in patients treated with CRS/HIPEC compared to palliative treatment [9]. Another study evaluating the benefit of elevated temperature in rats demonstrated that normothermic intraperitoneal chemotherapy was sufficient to eliminate tumor cells after CRS [10]. Altogether, clinical trials evaluating new treatment options for PM are scarce, and the concept of CRS/HIPEC invented by Paul Sugarbaker has not changed much over the years, resulting in the use of the same reagents for the last 20 years [6, 11].

Current research lacks a suitable human experimental *ex vivo* model to investigate new therapeutic reagents for HIPEC, as well as the mechanisms underlying PM formation. Existing *ex vivo* models include inverted bovine urinary bladders used to optimize intraperitoneal drug delivery [12], mouse peritoneum as a scaffold for human tumor cells [13], combined models of human mesothelial and fibroblast cells with rat extracellular collagen matrices [14] as well as mere human models from healthy donors [15], [16], [17] or artificial models [18], [19], [20]. However, these animal or combined models do not fully reflect the tumor biology including the tumor microenvironment in humans. Thus, new translational research models are urgently needed to investigate and compare alternative treatment options for PM.

Here, we established and characterized a human *ex vivo* peritoneal coculture model to mimic and investigate PM using either CRC cell lines or patient-derived tumor organoids. Furthermore, this model system was used to analyze possible treatment options for PM, such as HIPEC.

## Materials and methods

### Cell culture

The colorectal cancer cell line HCT116 was obtained from Jens Schmid (Dr. Margarete Fischer-Bosch Institute for Clinical Pharmacology, Stuttgart, Germany). HT29/GFP-luciferase cells were obtained from Julia Beil (University Hospital Tübingen, Eberhard Karls University, Internal Medicine VIII, Tübingen, Germany). Cell lines were authenticated by STR analysis (Eurofins, Ebersberg, Germany). For quality control, mycoplasma PCR using the Venor^®^GeM Classic kit (11-1025, Minerva Biolabs, Berlin, Germany) was performed according to the manual and before the use of cell lines. HCT116 cells were cultured in RPMI 1640 w/Glutamax (Thermo Fisher Scientific GmbH, Waltham, Massachusetts, US) supplemented with 1% penicillin/streptomycin (Biochrom AG, Berlin, Germany) and 10% FCS (Thermo Fisher Scientific GmbH, Waltham, Massachusetts, US) in a 5% CO_2_ incubator at 37 °C. HT29/GFP-luciferase cells were cultured in McCoy’s 5A Medium w/l-glutamine (Pan Biotech GmbH, Aidenbach, Germany) supplemented with 1% penicillin/streptomycin and 10% FCS in a 5% CO_2_ incubator at 37 °C. For coculture experiments 2,500–5,000 cells were seeded onto the tissue. After 24 h medium replaced with fresh medium to remove cells that did not attach to the tissue.

### Organoid culture

Patient-derived colorectal cancer organoids were generated and cultured as described earlier [21, 22]. In brief, the tissue was cut into small pieces and dissociated at 37 °C. Dissociated cells were passed through a 30 and 100 μm cell strainer and collected in advanced DMEM/F12 medium (Thermo Fisher Scientific GmbH, Waltham, Massachusetts, US) supplemented with Glutamax (Thermo Fisher Scientific GmbH, Waltham, Massachusetts, US), penicillin/streptomycin, HEPES, N-acetylcysteine (Merck Chemicals GmbH, Darmstadt, Germany), N-2 supplement (Thermo Fisher Scientific GmbH, Waltham, Massachusetts, US), B-27 supplement (Thermo Fisher Scientific GmbH, Waltham, Massachusetts, US), EGF (PeproTech GmbH, Hamburg, Germany), Y27632 (Absource Diagnostics GmbH, Munich, Germany), and amphotericin (Sigma-Aldrich Chemical, St. Louis, Missouri, US) and embedded in matrigel (Corning B.V., Amsterdam, Netherlands). For subcultivation, organoids were removed from matrigel and dissociated into small organoids using TrypLE (Thermo Fisher Scientific GmbH, Waltham, Massachusetts, US) and then transferred into fresh matrigel. For coculture experiments, organoids were dissociated into small organoids and 2,500–5,000 small organoids were seeded onto the peritoneum.

### Peritoneal culture

Residual tissue from patients with gastrointestinal tumors without peritoneal metastasis undergoing major elective surgery was collected after informed consent. Fresh peritoneal tissue was directly obtained from the surgery room and immediately transferred to the laboratory in E199 medium (Biochrom AG, Berlin, Germany). Afterward, the tissue was incubated for 15 min in PBS containing penicillin/streptomycin and amphotericin (Sigma-Aldrich Chemie, St. Louis, Missouri, US). Extraperitoneal fat was carefully removed with a scalpel, and small tissue pieces were inserted between two stainless steel rings and cultured with the mesothelial cell surface pointing upward. The tissue was cultured in E199 medium containing penicillin/streptomycin, L-glutamine (Biochrom AG, Berlin, Germany), FCS (Thermo Fisher Scientific GmbH, Waltham, Massachusetts, US), hydrocortisone (Sigma-Aldrich Chemie, St. Louis, Missouri, US), FGF (PeproTech GmbH, Hamburg, Germany), and heparin (Biochrom AG, Berlin, Germany) as described previously [16]. For fresh-frozen sections, the tissue was carefully dislodged from the rings, embedded in Tissue Freezing Medium (Leica Microsystems, Wetzlar, Germany) and immediately frozen in liquid nitrogen. For FFPE sections, the tissue was fixed with 4% PFA (VWR International, Radnor, Pennsylvania, US) and embedded in paraffin.

### Decellularization of peritoneal tissue

For decellularization, the peritoneal tissue was processed as described before and inserted between two stainless steel rings. Afterward, the tissue was incubated with buffer A (10 mM Tris, 0.1% EDTA, pH 7.8) for 18 h at 37 °C. The next day, buffer A was removed, and the tissue was washed twice with PBS, followed by incubation with 0.1% SDS for 24 h at 37 °C. Thereafter, the tissue was washed three times with buffer B (10 mM Tris, pH 7.8) and digested with digestion buffer (50 U/mL DNAse, AppliChem GmbH, Darmstadt, Germany, in 20 mM Tris, 2 mM MgCl_2_, pH 7.8) for 3 h at 37 °C. Decellularization was confirmed by H&E staining.

### *Ex vivo* hyperthermic treatment

Chemotherapeutic drugs were obtained from the in-house clinical pharmacy department, at 2 mg/mL doxorubicin-HCl (06581630, Teva GmbH, Ulm, Germany) and 1 mg/mL MMC (11213532, medac, Wedel, Germany) stock solutions. For HIPEC treatment, the peritoneal tissue was treated with 10 µM doxorubicin or 10 µM MMC diluted in culture medium for 90 min at either 37 °C or 41 °C in a 5% CO_2_ incubator. Afterwards, the tissue was washed gently with PBS and medium containing doxorubicin or MMC was replaced by normal culturing medium. For immunofluorescent evaluation of doxorubicin entry into peritoneal cells, the tissue was embedded immediately after treatment and fresh-frozen tissue sections of 3 µm were stained with Vectashield with DAPI (BIOZOL Diagnostica GmbH, Eching, Germany) and analyzed by a Leica TCS SP8 fluorescence microscope. For analysis of cell survival three days after hyperthermal treatment, the peritoneal tissue was digested with 2 mg/ml collagenase (Sigma-Aldrich Chemie GmbH, St. Louis, Missouri, US) for 24 h and the luminescent signal of HT29/GFP-luciferase cells was measured using a Bright-Glo™ Luciferase Assay System (Promega, Madison, Wisconsin, US) according to the manual.

### Immunohistochemistry

FFPE sections of peritoneal tissue (4 µm) were stained with Mayer’s hematoxylin (Sigma-Aldrich Chemie, St. Louis, Missouri, US) and eosin (Merck Chemicals GmbH, Darmstadt, Germany). After heat-induced epitope retrieval at pH 6 (CD3, CD68), pH 9 (vimentin, CD19, WT1) or proteinase K treatment (EpCAM), antibody staining (CD3: 1:200, MRQ-39, Lot-no. 0000052880, Cell Marque, Rocklin, US; CD19: 1:50, MRQ-36, Lot-no. 0000022072, Cell Marque, Rocklin, US; CD68: 1:8000, Kp-1, Lot-no. 1326701B, Cell Marque, Rocklin, US; EpCAM: 1:50, 248M-96, Cell Marque, Rocklin, US; Ki-67: 1:100, clone SP6, monoclonal rabbit IgG, Order-no. 275R-16, LOT-no. 0000075544, Cell Marque, Rocklin, California, US; vimentin: 1:200, V9, Lot-no. 0000036006, Cell Marque, Rocklin, US; WT1: 1:200, 6F-H2, 348M-96, Cell Marque, Rocklin, US) was performed for 25 min on a Lab Vision Autostainer (Thermo Fisher Scientific GmbH, Waltham, Massachusetts, US).

For extracellular matrix (ECM) staining, the following antibodies and pretreatments were used: Collagen I (ab34710, Abcam, Cambridge, UK, 1:100, no epitope retrieval), collagen III (ab6310, clone FH-7A, Abcam, Cambridge, UK, 1:150, heat-induced epitope retrieval at pH 6), collagen IV (M0785, Agilent, Santa Clara, CA, US, 1:200, pronase-induced epitope retrieval), MMP9 (NCL-MMP9-439, clone 15W2 Novacastra via Leica Biosystems, Wetzlar, Germany, 1:50, heat-induced epitope retrieval at pH 9). After antibody-specific epitope retrieval (Pronase 1 g/l, 107433, Merck, Darmstadt, Germany), endogenous peroxidase blocking (Dako, S2023) was performed for 10 min at room temperature. Primary antibody staining was performed at 4 °C overnight, followed by peroxidase/DAB+-based detection using the Dako REAL EnVision Detection System (Dako, K7005). Stainings were evaluated with the help of a trained pathologist from the Department of Pathology at the Robert Bosch Hospital.

### Transmission electron microscopy

Electron microscopy (EM) images were obtained in collaboration with the Institute of Pathology, University Hospital Tübingen, Germany. Tissue samples were fixed with 2.5% glutaraldehyde (Science Services, Munich, Germany) in cacodylate buffer (Merck, Darmstadt, Germany) at 4 °C overnight. Thereafter, samples were embedded in araldite (Serva, Heidelberg, Germany), and ultrathin sections were cut using a Leica ultramicrotome (Leica, Wetzlar, Germany). Sections were analyzed in a Zeiss EM-10 transmission electron microscope (Zeiss, Oberkochen, Germany).

### Statistics

Statistical analyses comparing control and treatment conditions were performed using a two-sided Student's t test (paired/unpaired as indicated in the figure legend) in Microsoft Excel 2016. All experiments are shown as the mean and standard error (SE) of at least three independent experiments.

## Results

### Establishment of a human *ex vivo* peritoneal model

**Figure 1:**
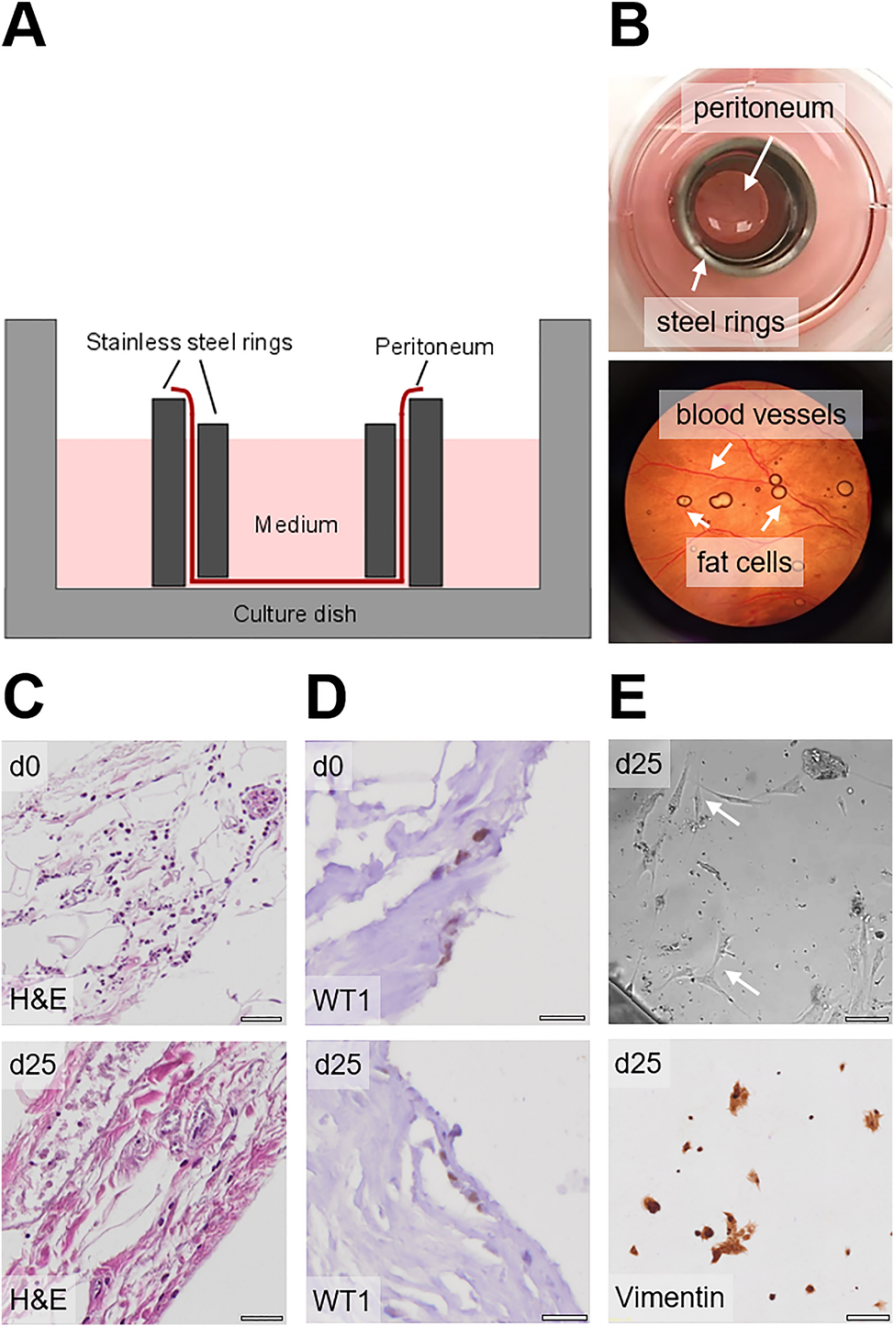
Establishment of a human *ex vivo* peritoneal model. (A) Schematic setup of the *ex vivo* peritoneal model. (B) Photographic images of the peritoneal model. (C) H&E staining of peritoneal tissue on day 0 and day 25 of culture; scale bars 200 µm. (D) WT1 staining of peritoneal mesothelial cells on day 0 and day 25; scale bars 20 µm. (E) Light microscopy and vimentin staining of outgrowing fibroblasts on day 25; scale bars 200 μm.

Here, we established and characterized a human *ex vivo* peritoneal model to investigate PM in a clinically relevant *ex vivo* system. Human peritoneal tissues were obtained from the surgery room and immediately transferred to the laboratory in sterile tubes. The tissue was washed with washing solution and PBS and subsequently inserted between two stainless steel rings for culture, as illustrated in Figure 1A and B. For characterization of the tissue and different cell populations within the peritoneum, FFPE blocks and tissue sections were cut before and after culture. H&E and immunohistochemical (IHC) stainings revealed the integrity of the tissue for up to 25 days of culture. Nucleic structures showed no alteration pointing to no change in viability of the peritoneal tissue (Figure 1C). WT1 positive mesothelial cells were detected for up to 25 days of culture (Figure 1D). In addition, as a sign of viability upon culture, vimentin-positive fibroblasts migrating out from the peritoneum were observed by light microscopy. These fibroblasts could be kept alive and further passaged in culture for up to 25 days (Figure 1E).

### Immunohistochemical characterization of peritoneal cells and ECM components

**Figure 2:**
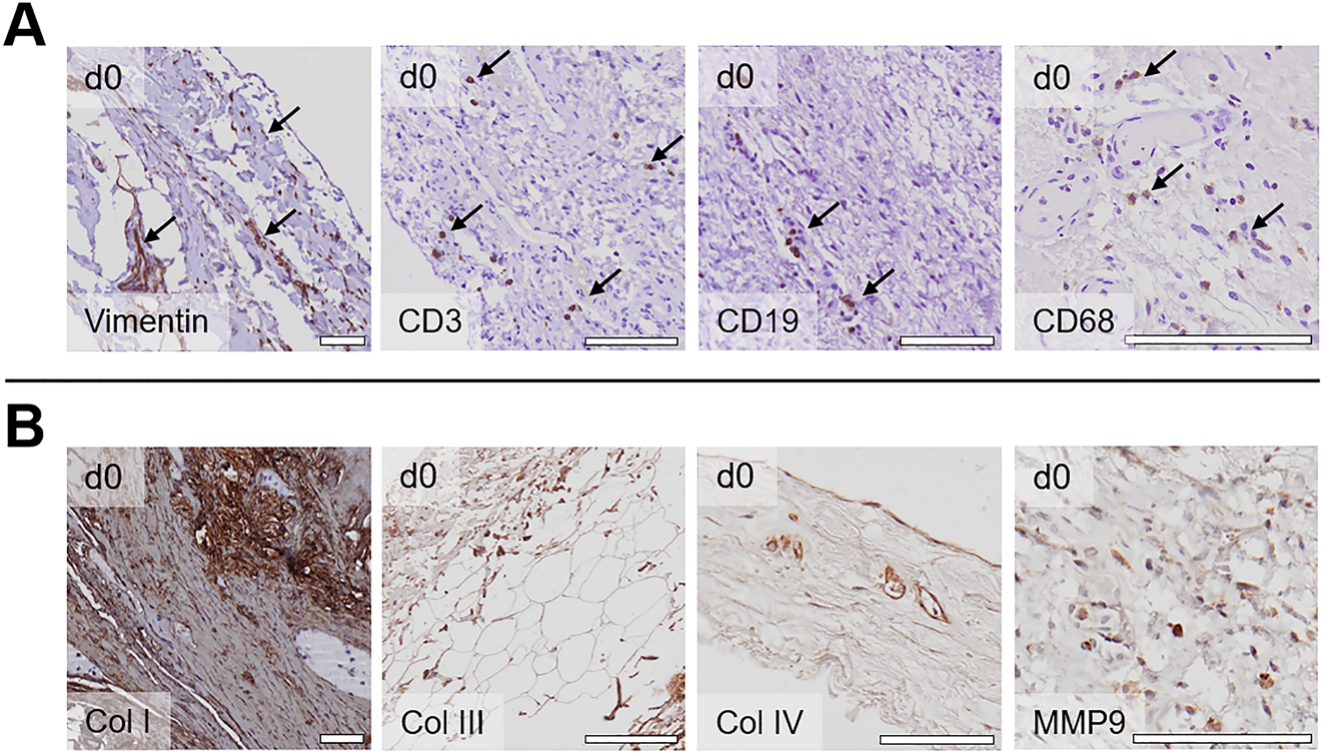
Immunohistochemical characterization of peritoneal cells and ECM components. (A) Staining for cellular markers before culture (day 0); vimentin: fibroblasts, CD3: T-lymphocytes, CD19: B-lymphocytes, CD68: macrophages; scale bars: 100 µm. (B) Staining for ECM components before culture (day 0); scale bars 100 μm.

In the next step, we analyzed the different cell populations within the peritoneum. As shown in Figure 2A, vimentin-positive fibroblasts and occasionally CD3-positive T-lymphocytes, CD19-positive B-lymphocytes, and CD68-positive macrophages were found in the tissue before culture (d0). Whereas CD3-, CD19- and CD68-positive cells could be detected for up to seven days in culture, vimentin-positive fibroblasts were found for up to 25 days. Fibroblasts were widely distributed in the tissue, while immune cells were most frequently found in proximity to vessels. Next, we investigated the distribution of extracellular matrix (ECM) components such as collagens and matrix metalloproteinases (MMPs): We found that collagen I and collagen III were strongly expressed and widely distributed in the peritoneum (Figure 2B), whereas collagen IV was specifically expressed around vessels and in the basal lamina underlying the mesothelial cells. In contrast, the ECM modulator MMP9 was only weakly expressed in the peritoneal cells of donors that did not suffer from PM.

### Establishment and characterization of a human *ex vivo* peritoneal coculture model

**Figure 3:**
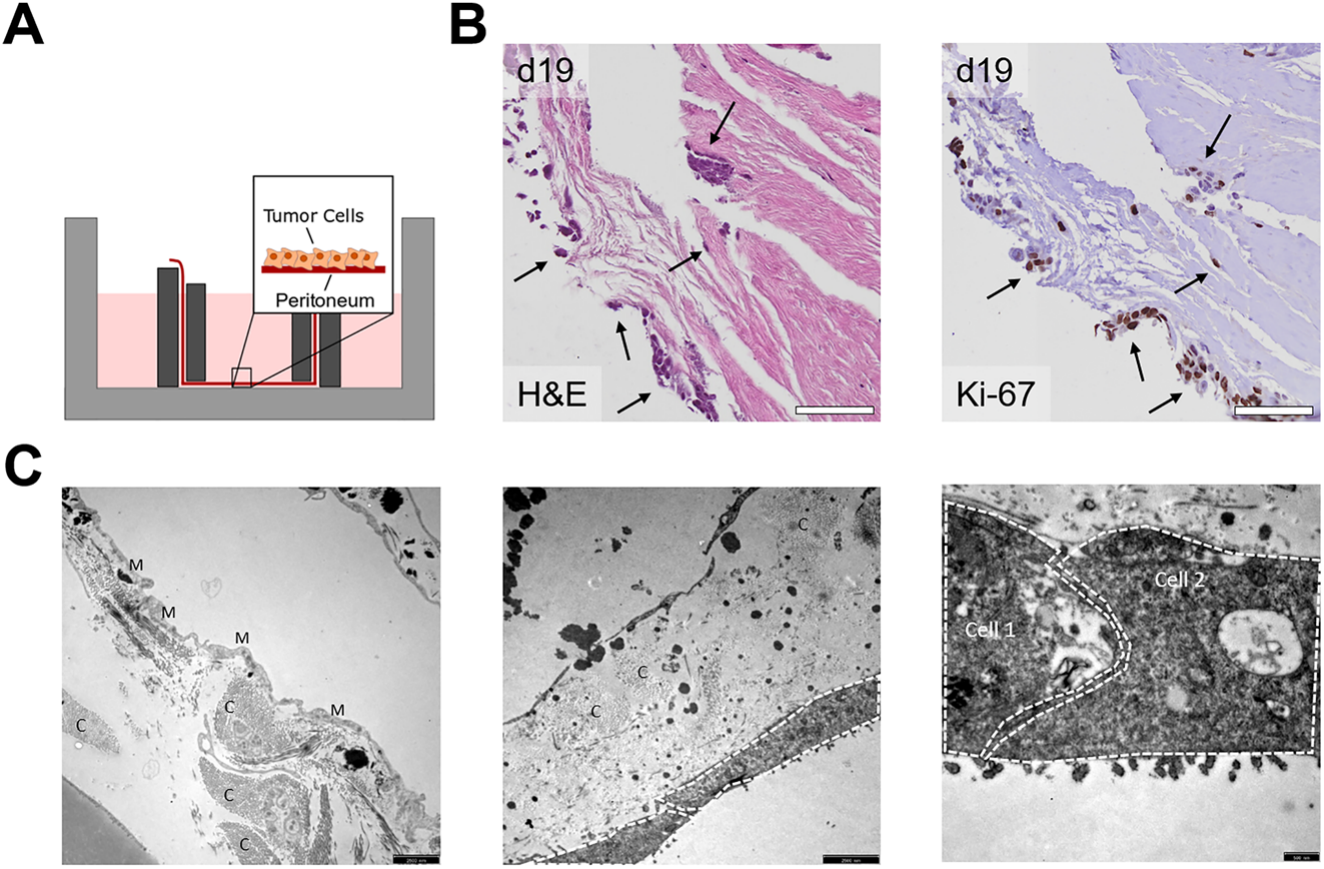
Establishment and characterization of a human *ex vivo* peritoneal coculture model. (A) Schematic setup of the *ex vivo* peritoneal coculture model. (B) H&E and Ki-67 staining for cocultures with the CRC cell line HCT116 at day 19 after culture; attached and invading CRC cells are indicated by arrows; scale bars 100 µm. (C) EM images of human peritoneum without (left) and with HT29 cells (middle, right, indicated by white lines) before culture (day 0, left) and day 6 after coculture (middle, right); M: mesothelial cells, C: collagen, Cell 1 and Cell 2: HT29 cells grown on the peritoneum; scale bars 2500 nm (left, middle) and 500 nm (right).

In the next step, we investigated whether peritoneal tissue could serve as an *ex vivo* model system for PM. As illustrated in Figure 3A, the peritoneal tissue was inserted between two stainless steel rings and cultured with the mesothelial cell surface pointing upward. A sufficient supply with medium to all parts of the tissue was assured at any time during culture. To mimic PM, CRC cell lines were added and cocultured with the peritoneum for up to three weeks. H&E and IHC staining for the proliferation marker Ki-67 showed that CRC cells attached to and invaded into the peritoneum and could be kept alive for up to 19 days (Figure 3B). To further characterize the peritoneal tissue as well as the coculture model, we performed EM imaging of peritoneal tissue before coculture (Figure 3C, left) and six days after coculture with the CRC cell line HT29 (Figure 3C, middle, right). The mesothelial cell layer was visible before culture (Figure 3C left) and ECM components, such as collagens, were present before culture and in coculture with HT29 cells (Figure 3C left, middle). After six days of coculture, HT29 cells (indicated by white lines) attached to and grew onto the peritoneum (Figure 3C, middle, right). The glycocalyx of HT29 cells was visible as small “bristles”.

### Modeling peritoneal metastasis using patient-derived organoids

**Figure 4:**
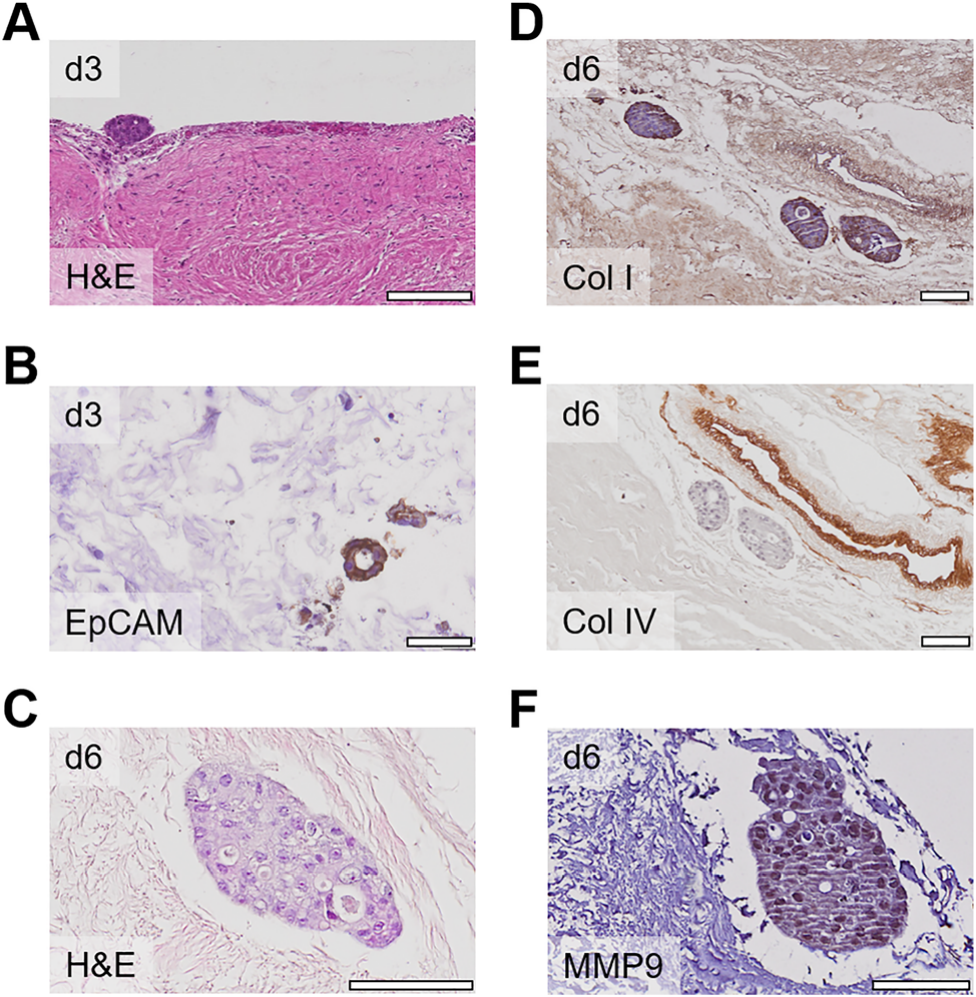
Modeling peritoneal metastasis using patient-derived organoids. (A) Patient-derived organoids of different donors attached to the peritoneal surface at day 3 after coculture; scale bar 200 µm. (B) Coculture of patient-derived organoids with cellularized peritoneal tissue after 3 days; sections were stained with EpCAM to distinguish between EpCAM positive tumor cells and negative peritoneal cells; scale bar 40 µm. (C–E) Coculture of patient-derived organoids with decellularized peritoneal tissue; after 6 days, sections were stained with H&E (C) and for collagen I (D), collagen IV (E), and MMP9 (F); scale bars 100 μm.

Next, we investigated whether our established *ex vivo* peritoneal model could serve as a scaffold for patient-derived organoids to model PM by CRC tumors. In the course of the disease or during surgery it is possible that small cell clumps or even complete glandulae in addition to single tumor cells get shed off from the primary tumor and metastasize to the peritoneum [4]. For this, patient-derived CRC organoids from five different donors were seeded on the peritoneum and cocultured for three days. The growth patterns were analyzed by H&E and EpCAM staining, showing that organoids attached to and migrated into the peritoneum (Figure 4A and B). To test, whether cell–cell contacts are pivotal for invasion and to investigate the role of ECM components in PM, we cocultured patient-derived organoids on decellularized peritoneal tissue. In this setting, the peritoneal cells were depleted from the tissue leaving only peritoneal ECM components such as collagen fibers. Patient-derived organoids were then seeded onto decellularized peritoneal tissue. After six days of coculture, FFPE sections were cut and stained for ECM components showing that organoids also invaded the peritoneum into deep peritoneal layers of decellularized tissue (Figure 4C). IHC stainings for ECM components revealed a diffuse and widely distributed positive staining for collagen I (Figure 4D), vessels that were positive for collagen I and IV, while organoid margins were only positive for collagen I (Figure 4D and E). Interestingly, organoids were strongly positive for matrix metalloproteinase 9 (MMP9), which is a matrix remodeling enzyme involved in epithelial–mesenchymal transition (EMT) and metastasis formation (Figure 4F). This shows that not only cell–cell interactions but also cell–ECM interactions are important for metastasis formation.

### Investigating the effects of hyperthermal treatment using a clinically relevant *ex vivo* peritoneal metastasis model

**Figure 5:**
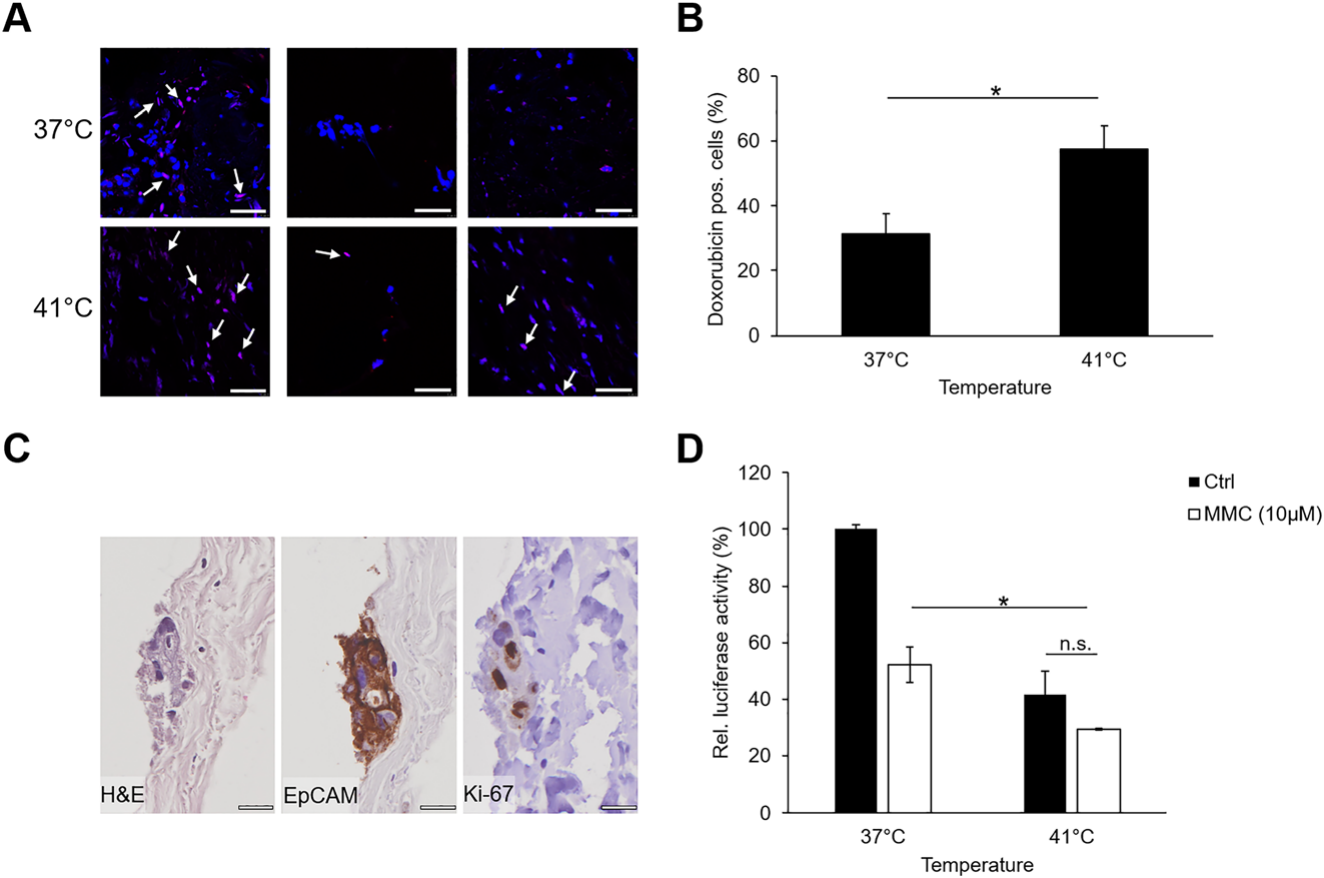
*Ex vivo* peritoneal model as a model system for hyperthermal chemotherapeutic treatment. (A) Doxorubicin immunofluorescence (red) of the *ex vivo* peritoneal model treated with 10 µM doxorubicin for 90 min at 37 °C or 41 °C; doxorubicin (red), nuclei (blue); white arrows indicate doxorubicin-positive cells in three representative images; scale bar, 40 µm. (B) Percentage of doxorubicin-positive cells following treatment at 37 °C or 41 °C (n = 3; two-sided, paired Student's t test: p = 0.007). (C) Coculture of HT29-GFP/luciferase cells with human peritoneum. EpCAM and Ki-67 stainings on day 6 reveal living and proliferating cancer cells; Scale bar 20 µm. (D) Treatment with 10 µM MMC for 90 min at 37 °C or 41 °C reduces the amount of living HT-29-GFP/luciferase cells 3 days after treatment; shown is one representative of at least three independent experiments performed in triplicates; significance was calculated using a two-sided paired Student’s t test; p=0.003.

PM of colorectal origin can be treated with HIPEC following complete resection of the tumor and peritoneal seeds. However, the clinical benefit of hyperthermia and the exact biological mechanisms are not yet fully understood. Therefore, we investigated whether our established *ex vivo* peritoneal coculture model could serve as a model system for peritoneal chemotherapeutic treatments such as HIPEC. To determine the influence of temperature on the availability of chemotherapeutic drugs within the peritoneum, we analyzed the percentage of peritoneal cells, which showed an orange fluorescence signal due to intranuclear doxorubicin accumulation following an incubation of the tissue with 10 µM doxorubicin for 90 min at 37 °C and 41 °C, respectively. Doxorubicin was used in this setting due to its intrinsic fluorescence. As shown in Figure 5B, hyperthermal treatment at 41 °C significantly enhanced the number of doxorubicin-positive nuclei from 31 to 58% (p = 0.007).

In further experiments, we examined whether hyperthermal treatment could reduce the survival of cancer cells cocultured with peritoneal tissue. For this, the amount of living cancer cells after treatment was determined by a light signal generated by luciferase-expressing HT29 cells. However, in this set up doxorubicin interfered with the experimental readout due to its intrinsic fluorescence and therefore MMC was used instead. As shown in Figure 5C, hyperthermal treatment for 90 min at 41 °C with 10 µM MMC significantly reduced the survival of HT29/GFP-luciferase cells within the peritoneum by 23% compared to treatment at 37 °C (p = 0.003). Thus, our *ex vivo* peritoneal model is suitable for modeling and investigating possible treatment options for PM.

## Discussion

In this study, we outlined that a human *ex vivo* peritoneal coculture model can be used to mimic the PM of CRC cell lines and patient-derived CRC organoids. This model can now be used to investigate current and future treatment options for CRC and PM, such as HIPEC.

The establishment and characterization of a human *ex vivo* peritoneal model was first shown by Falk et al., who used this model to study mesh-tissue integration in hernia surgery [16]. In this study, we further developed and characterized the peritoneal model, showing that patient-derived peritoneal cells could be kept alive in culture for up to 25 days, including the critical mesothelial cells, which was evident by the integrity of the tissue and nucleic structures in H&E and IHC stainings over time, as well as fibroblasts migrating out from the peritoneum. In addition, we observed that fibroblasts, which had migrated out from the peritoneum, could be kept alive and further passaged in culture for at least two weeks. Moreover, we showed that the human *ex vivo* peritoneal model is comprised of different cell types, including fibroblasts and immune cells, such as CD3-positive T-lymphocytes. However, additional studies will be needed to further clarify the interaction between these resident immune and cancer cells in our *ex vivo* model.

Although we noticed some discrepancy to other published *ex vivo* models using human tissue regarding the viability of the tissue and the integrity of the mesothelial cell layer *ex vivo* [15], integrity and survival of peritoneal cells during *ex vivo* culture was comparable to previous publications [16]. Using human residual tissue from different donors results in a batch-to-batch variability and may contribute to the discrepancy seen, but also differences in transport media composition and handling time between tissue removal and arrival in the lab may cause a significant disparity in viability and tissue integration.

In the present model, tumor cells and patient-derived tumor organoids not only attached to but also migrated into the peritoneal tissue. This is an important observation with potential clinical impact, as it shows that tumor cells that are shed off from the primary tumor could attach to and grow into injured sites of the peritoneum, which was already seen in animal models [23], [24], [25], [26]. In accordance with that, previous investigations observed a tendency of colorectal tumor cell lines to adhere to traumatized sites of the peritoneum or *ex vivo* cultured human mesothelial cells [27].

To address the question if and to which extent peritoneal cells and ECM components are involved in PM, we used the *ex vivo* model in a different set-up: Peritoneal cells were depleted from the tissue and the remaining scaffold consisting of ECM components only was cocultured with patient-derived organoids. We here detected an infiltration of organoids into the tissue indicating that cell–cell interactions between tumor and peritoneal cells might not be the only prerequisite for seeding. In addition, we investigated the distribution of ECM components showing that MMP9, which is involved in matrix remodeling during metastasis, was highly expressed in invading organoids. Similar approaches investigating the influence from the surrounding tumor microenvironment on primary CRC and breast cancer development were recently established using decellularized colon and breast tissue, respectively [28], [29], [30]. Therefore, this coculture model is a useful tool to mimic cancer cell attachment and invasion during PM. We here observed a positive immunoreaction for collagen I but not collagen IV around organoids which was uncommon but not unexpected: Although matrigel mainly consists of collagen IV, there seems to be a batch-to-batch variability in the composition that was already shown by other groups [31, 32].

Patients with PM have a very poor prognosis [5] and the molecular mechanisms behind PM originating from CRCs are poorly investigated. Moreover, clinically relevant models to investigate PM and possible treatment options apart from animal models are still scarce [15, 17, 33, 34]. Recently Asano and colleagues developed an artificial human peritoneal tissue (AHPT) model to investigate PM of different cancer types [18], [19], [20]. This bottom-up approach consists of a mesothelial cell layer resting on several layers of human fibroblasts and tubular arranged endothelial cells connected via the cell-accumulation technique [34]. This artificial model provides a reproducible platform for basic research but is time consuming and requires an additional expertise regarding the preparation of the model in advance for any experimental coculture set-up compared to our peritoneal model that is based on residual tissue from human donors. Moreover, by using human residual tissue as basis, we included interindividual differences in tissue architecture, making this model more robust and comparable to the real in human situation. Thus, our established human *ex vivo* coculture peritoneal model using CRC cell lines and patient-derived CRC organoids helps to investigate the biology of PM and possible treatment options.

HIPEC is a current treatment option for patients with PM that is performed after complete resection of the primary tumor and all visible metastases. However, the molecular mechanisms and benefits of hyperthermal chemotherapy are controversial and not fully investigated [10, 35, 36]. This is, to our knowledge, the first study investigating HIPEC in a human *ex vivo* peritoneal model. Previous studies by Schaaf et al. already showed that penetration of doxorubicin into the peritoneum was enhanced upon hyperthermia [37]. Here, we used similar concentrations, temperatures, and times for HIPEC treatment of patient-derived peritoneal cells and monitored the entry of doxorubicin into the cells by fluorescence microscopy: Hyperthermia significantly enhanced the entry of doxorubicin into the peritoneum. In addition, hyperthermal treatment with MMC was more effective in killing HT29 cells than MMC treatment at 37 °C thus showing a clear benefit of higher temperatures for intraoperative chemotherapy. Although the combination of chemotherapy and hyperthermal treatment had a significant impact on cell survival, we did not aim to optimize these treatment conditions in this set of experiments, rather showing the applicability of the model to investigate possible treatment options. Investigating optimal treatment conditions will be part of future studies.

In this study, we established an *ex vivo* peritoneal coculture model with CRC cell lines or patient-derived organoids that provides a stringent and clinically relevant platform for the investigation of different players during PM and for the elucidation of possible treatment options. Future research based on this model could not only elucidate factors contributing to the formation of PM but also contribute to the development of tailor-made medical and surgical therapies.
